# Considering the Impact of Social Media on Contemporary Improvement of Australian Aboriginal Health: Scoping Review

**DOI:** 10.2196/11573

**Published:** 2019-02-05

**Authors:** Troy Walker, Claire Palermo, Karen Klassen

**Affiliations:** 1 Be Active Sleep Eat Department of Nutrition, Dietetics and Food Monash University Notting Hill Australia

**Keywords:** aboriginal, Indigenous, social media, health, Web-based

## Abstract

**Background:**

Social media may have a significant role in influencing the present and future health implications among Australian Aboriginal and Torres Strait Islander people, yet there has been no review of the role of social media in improving health.

**Objective:**

This study aims to examine the extent of health initiatives using social media that aimed to improve the health of Australian Aboriginal communities.

**Methods:**

A scoping review was conducted by systematically searching databases CINAHL Plus; PubMed; Scopus; Web of Science, and Ovid MEDLINE in June 2017 using the terms and their synonyms “Aboriginal” and “Social media.” In addition, reference lists of included studies and the Indigenous HealthInfonet gray literature were searched. Key information about the social media intervention and its impacts on health were extracted and data synthesized using narrative summaries.

**Results:**

Five papers met inclusion criteria. All included studies were published in the past 5 years and involved urban, rural, and remote Aboriginal or Torres Strait Islander people aged 12-60 years. No studies reported objective impacts on health. Three papers found that social media provided greater space for sharing health messages in a 2-way exchange. The negative portrayal of Aboriginal people and negative health impacts of social media were described in 2 papers.

**Conclusions:**

Social media may be a useful strategy to provide health messages and sharing of content among Aboriginal people, but objective impacts on health remain unknown. More research is necessary on social media as a way to connect, communicate, and improve Aboriginal health with particular emphasis on community control, self-empowerment, and decolonization.

## Introduction

The need for evidence-based strategies to improve the health of Australia’s Aboriginal and Torres Strait Islander people (hereafter referred to as Aboriginal) is essential to bridging the 10-year gap in life expectancy [1]. Addressing behaviors perpetuating chronic disease linked to diet and lifestyle and the underlying determinants is complex and, thus, require multifaceted solutions, including screening, assessment, and treatment, support for behavioral change, and changes to the environment to promote healthy choices [2,3]. Having Aboriginal communities at the center of the design and delivery of health-related programs is well established to improve outcomes [4,5].

Social marketing applies marketing principles to disease prevention programs to facilitate health behavioral change [6]. An increase in the use of the internet and portable devices and apps has increased the use of social media as an avenue for social marketing. Social media is any Web-based communication dedicated to participant-based input, interaction, content-sharing, and collaboration. Social media is increasingly being used to try and improve health across the entire population. A recent systematic review of the use and advantages of social media for health communication identified its increasing use and potential to improve health outcomes. Yet, the literature on its benefits and application in Aboriginal populations has not been systematically explored; understanding its potential to improve health in Aboriginal populations is important as the use of some social media in remote areas has been reported as 20% higher than the national average [7]. In addition, some evidence suggests that much of the media portrayal of Aboriginal people is negative and may lead to poorer health outcomes [8]. Racial vilification, where the collective trauma of Aboriginal people is publicized, triggers painful reminders of colonialism [9]. Moreover, sexually explicit content is readily available to the youth of illegal age [3]. However, other data suggest that the ability of social media to support the creation and sharing of content and networking provides opportunities for health messages to be conveyed to a wider social network [10]. While there is some evidence to support the role of social media to promote and improve health in Aboriginal people, there is little evidence of its effect.

Of the evidence that exists, it appears social media may provide a contemporary conduit for Aboriginal people’s expression of culture and the ability to access novel ways of health-related knowledge, learning, and engagement among one another and the wider community [11]. Little evidence includes the impact or effect of social media to change behavior or cultural norms [12]. Thus, there is a need to investigate the role of social media in delivering messages related to health for Aboriginal people and its impact on health outcomes.

This study aims to examine the extent of health initiatives using social media that aimed to improve the health of Australian Aboriginal communities.

## Methods

### Study Conception

To conceptualize outcomes relative to our question, we undertook a scoping review of the potential breadth of health implications that social media may have on Aboriginal Australian’s health and well-being. A systematic approach, informed by the Preferred Reporting Items for Systematic Reviews and Meta-Analyses guidelines and Population, Intervention, Comparison and Outcomes, was used to construct the research question and search terms [13,14]. A scoping review approach was selected on the basis of the paucity of evidence in the field; this methodology was chosen to summarize what was known and identify gaps based on the guidelines of scoping reviews developed by Arksey and O’Malley [15] and advanced by Levac [16].

Our methodology included searching and reviewing the literature to examine the extent and type of work being undertaken in relation to using social media to improve health in Australian Aboriginal communities and summarize this work and identify gaps. A team of 3 researchers were involved in the totality of the process, 1 Aboriginal Australian and 2 non-Aboriginal Australians.

### Search Strategy

The databases CINAHL Plus, PubMed, Scopus, Web of Science, and Ovid Medical Literature Analysis and Retrieval System Online were searched in June 2017 using the terms and their synonyms “Aboriginal” and “Social media” (Textbox 1); this was followed with citation snowballing from relevant systematic reviews and included full-text papers whereby reference lists were scanned. In addition, the first 5 pages of the Aboriginal health evidence repository website Australian Indigenous Health InfoNet was searched for gray literature in September 2017 using the same search terms.

### Data Management

In August 2017, results were exported to Covidence software (Covidence Systematic Review Software, Veritas Health Innovation) [17]. Titles and abstracts from searchers were screened by 2 authors (TW and CP). Differences of opinion were resolved through consensus discussions, and where agreement could not be reached, a third author (KK) was brought in to resolve.

### Inclusion and Exclusion Criteria

All study designs were included. Published and unpublished studies were included; however, guidelines, protocols, opinion pieces, conference abstracts, and review papers were excluded. Systematic reviews were excluded; however, the reference lists of these studies were searched for relevant papers. Included studies must have reported participants who identified as Aboriginal or Torres Strait Islander, as well as some form of evaluative judgment on the role of social media on improving health.

The intervention or phenomena of interest was social media. Outcomes of interest were the acknowledgment, betterment, or detriment of Aboriginal-related health. In this instance, Aboriginal health and well-being were defined as any potential effects that improved or impaired any element of health, recognizing Aboriginal people’s broad conceptualization of health [18]. Papers were excluded where studies were not specific to health outcomes in Aboriginal people and, concomitantly, where there was an absence of the use of social media in conjunction with a focus on improving health outcomes.

### Data Analysis

Data extracted included author, date, location, sample size, and demographics (if known), as well as interventions, potential outcomes, and findings related to the study aims. In addition, notes on whether there was Aboriginal involvement in the study were recorded and the impacts of social networking sites on Australian Aboriginal health and well-being specifically summarized.

**The list of databases and all search terms used undertaken in June 2017.**

**Databases used:**

- Ovid Medical Literature Analysis and Retrieval System Online
- Scopus
- CINAHL Plus
- PubMed
- Web of Science

**Search terms used:**

- aborigi* OR indigen* OR “oceanic ancestry group” OR “first australian” OR “Torres strait islander*” AND
- “social media” OR “social networking site” OR “social network* website*” OR “online social network” OR “online network” (Truncation)

Owing to the small number of included studies, this information was used to understand where the evidence currently exists and inform gaps, rather than a synthesis of findings. Narrative summaries of the qualitative studies included comparing and contrasting social media interventions across studies, as well as potential outcomes from each study to assist in informing a summary of the role of social media in improving the health in Australian Aboriginal communities as is typical of scoping reviews.

### Quality Assessment

Studies were assessed for quality by 2 authors (CP and TW) using the Critical Appraisal Skills Programme tool [19]. Studies were scored out of 8 criterion points based on the quality assessment. Studies with a score of >4 (out of 8) were considered good quality; studies with a score of 4 were considered neutral quality and studies with a score of <4 were considered poor quality.

## Results

### Study Criteria

**Figure 1:**
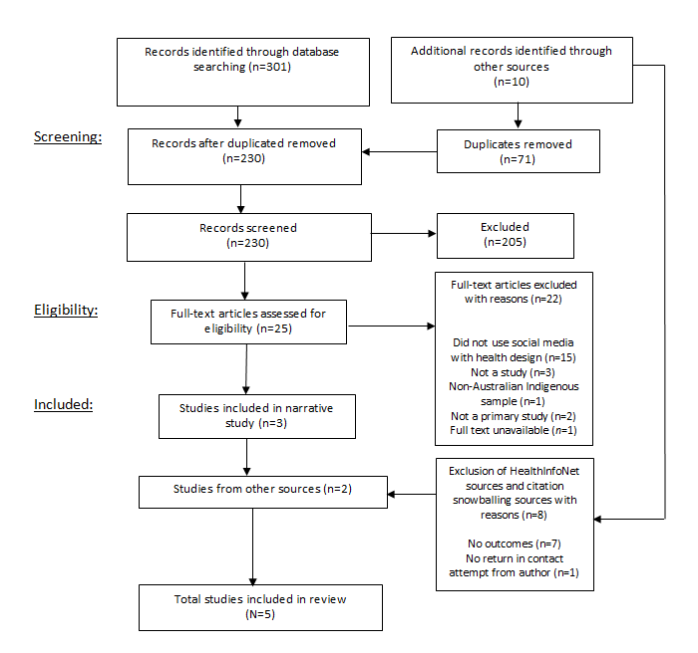
The study selection flow diagram representing the selection of studies included in the systematic literature review.

The initial search revealed 301 studies, which after duplicates were removed, leaving 234 for screening (Figure 1). Screening 234 titles and abstracts left 25 full-text papers for full-text screening. The full text of the remaining 25 papers was assessed, and a further 22 papers were excluded because of not meeting both health-related and social media-related outcomes, or because of not being an empirical study where some evaluation of the intervention was undertaken. Citation snowballing found an additional 7 papers, and following full-text assessment, 1 was included. Searching the gray literature in HealthInfoNet produced 3 papers relevant for assessment. Of the 3 included, 2 more were excluded; 1 was excluded owing to the inability to retrieve further information through contact with the author, whereas the second was excluded given the absence of health and related outcomes.

### Included Study Features and Quality Assessment

While only 5 studies were found, a narrative summary of these studies was deemed appropriate to assist in understanding the role of social media in improving the health of Aboriginal Australians and guide future research in this area. Of the 5 included papers, 3 used qualitative approaches [11,20,21] and 2 used multiple methods [22,23]. Based on the quality assessment tool used, from the 5 studies, 2 were of neutral quality, 2 were of poor quality, and 1 was of good quality (Multimedia Appendix 1).

All papers were published within the past 5 years, from 2013 to 2017. The studies involved Aboriginal people aged 12-60 years and from both males and females. The settings of the social media campaigns were initiated in urban, rural, and remote Australian locations where they allowed for more widespread involvement. Study durations ranged from 1 day (15 hours) to the present day with ongoing reporting. Participant numbers varied between 28 and 346 people. A key feature and strength of all studies were that Aboriginal people were part of the research project and either involved in gathering experiential data, forming researcher-community partnerships or the research writing itself [24]. No studies reported objective impacts on health.

Social media was used as a tool to enhance social support in all studies, whereby community members were connected online. Social support occurred between social media users or users and Aboriginal health organizations by linking real-world events with Web-based conversations and in improving awareness of access to offline social and emotional support. In addition, social media was used to disseminate information more widely outside the study population as social media was proposed to provide a platform for reaching a broader audience.

One study found that age was associated with social media use for health [21]. Older Aboriginal groups often found using social media for health more complex and, in some cases, having detrimental health outcomes [21], whereas younger groups were more readily receptive to using social media for their health and well-being [11,20,21]. All studies mentioned the need for more time for participants to become familiar with utilizing social media for it to have an impact on health; the reason acknowledged was the relative infancy of social media use and Aboriginal health within Australia [21,23]. All studies showed improved health, which included exercise, nutrition, family, mental health, suicide, death, and grieving. Furthermore, all outlined the need for future social media health campaigns to consider current Australian Aboriginal health culture and perspective [11,20-23].

A repeated theme that appeared in 3 papers was that social media provided greater space for sharing health messages in a 2-way exchange [11,20,23]. One paper noted the increased awareness and self-empowerment of Aboriginal people in governing their own health after applying one particular social media campaign [11]. Another study showed that when the aim was to increase the quality and duration of Aboriginal people’s lives, an emphasis on sport and promotion of physical activity using social media as a medium was well received based on the overall participation and positive feedback. When this approach was combined with other health behaviors, such as quitting smoking, or decreasing the consumption of added sugar and sugar-sweetened beverages, more positive qualitative responses were apparent [22].

Negative health impacts were described in 2 papers using social media, where it was perceived to represent Aboriginal people in a poor light relative to health-related conditions [11,20]. One study specifically noted it could be inadvertently disrespectful by displaying death notices where elder Aboriginal people were unable to use or access social media; this included learning of illness, deaths, and funeral services belatedly in the family through Facebook rather than in-person [21]. Another study outlined that the consistent focus on the health implications, including chronic diseases like diabetes, obesity, and mental health, was potentially negative and labeled Aboriginal people into a deficit position [20]. Respondents in these 2 studies voiced concern with the negative images portrayed in all forms of media of Aboriginal people regarding their health [11,20].

## Discussion

### Principal Findings

This study aimed to examine the extent of health initiatives using social media that aimed to improve the health of Australian Aboriginal communities; it found 5 studies that evaluated the impact of a range of social media strategies on health or well-being. Social media provided a space for providing social support, sharing health-promoting messages, and increasing awareness and self-efficacy of Aboriginal people in governing their own health. The cocreation of social media content with Aboriginal people and concepts of both self and community empowerment that aimed to improve health appeared to be well received based on the participation and positive feedback.

Literature is scarce regarding the use of social media as a conduit in promoting the health of Australian Aboriginal people. To the best of our knowledge, this is the first scoping review using a systematic approach to evaluating the evidence of health initiatives using social media that aimed to improve the health of Australian Aboriginal communities. A consistent and apparent theme was the concept of a healing and self-empowering dialogue among Aboriginal people. These themes, while often termed in a variety of different ways, centered around end users, researchers, and funders working together to construct contemporary ways to refine, expand, and improve Aboriginal health using multiple platforms of social media. Common alternate names used included, but were not limited to, cocreation, self-determination, 2-way communication, and self-design [11,20,25,26]. Most studies focused on the positive elements of improving Aboriginal health; this is in contrast to much of the previous literature, which framed their research around “disease” and the problems associated with the disease rather than “health.” Other work has investigated social media and its role in racial vilification [9]. The examples analyzed in this study show that social media has significant negative and detrimental impacts on Aboriginal people as they are reminded of colonization. However, the authors acknowledge that their findings highlight the potential vehicle of social media to have conversations that promote change [9]. In addition, a recent study has found that Australian Aboriginal people interact about their health using social media [27]. Our review highlighted that research that addresses and evaluates decolonization and self-empowerment will be more likely to improve Aboriginal health outcomes [11,20,28]. Sharing health information online may gather traction and community capital among Aboriginal communities when using positive messages related to diet, exercise, or smoking rather than threatening approaches frequently used in health media campaigns [27]. When there is an online sense of community support, with a particular focus on self-empowering language that promotes and encourages making better choices related to Aboriginal health and well-being, participation in social media may increase; this area shows promise for more work, given its positive reception and popularity among Aboriginal people [11]. More evaluation is warranted with framing “health” positively to improve Aboriginal health and its associated outcomes.

Social media was used as a platform for social support in most of the included studies. As social and emotional well-being and community connectivity are important for Aboriginal people, enhanced access to social support networks is important for enabling behavioral change [20,29-31]. Social media, through its increased reach could enhance and enlarge support networks; this is important for all Australian Aboriginal communities where access to support may be limited. In addition, information dissemination of public health messages and increasing awareness of access to support and health care can be enhanced for those living in remote communities [32]. The unfavorable findings within the included studies was that social media could be perceived to represent the health of Aboriginal people negatively [20,21,23] or conjure up emotion when learning about funeral services, death, and grieving on social media rather than in-person [21]; these are important considerations for the future use of social media in Aboriginal communities. Likewise, other work has shown that social media may heighten and increase conflict and violence among feuding families [33]. As social media can be used to increase reach for health messages, it can also be used to amplify stigma, racism, and bullying by more widely spreading negative messages. Social media can be used to propagate stigma, and this has been observed in many stigmatized health conditions such as mental health and Alzheimer’s disease [34,35]. Important lessons were learned from #IHMayDay social media strategy as concerns were prospectively raised about the detrimental impacts of negative framing and participants were urged to engage positively throughout the day [20]; this negative potential of social media must be considered for future interventions.

Respecting and appreciating traditional customs of Indigenous groups in building scientific evidence for Indigenous people has been called for in other work [36-38]. The impact of racism on psychological health and the overall negative approach taken by the portrayal of Maori people in all forms of media has been previously highlighted [38]; this fault is noted as a result of the adaptation to recent colonization. A recent systematic review of social marketing targeting Indigenous people across the world found that social marketing interventions primarily used television and radio advertising and appeared to confront health issues of Indigenous populations around the world despite not maximizing all elements of social marketing [39]. These findings together provide evidence for the need to consider social media as strategies to improve the health of Australia’s Aboriginal people, acknowledging the need to use positive health messaging and portray these communities using a strengths-based focus.

### Limitations

This study is limited to social media and does not include other social marketing campaigns. Studies only focused on Australian Aboriginal populations and may not be relevant to other Indigenous populations across the world using other platforms for social marketing beyond social media. However, this scoping review has highlighted the lack of studies that actually examine the impact of health-related social media activities in Aboriginal people. While inferences are made toward the perceived or self-reported impact on health or well-being, there was no actual objective measurement in any of the included studies. There is a need for work examining the impact of social media on actual health outcomes.

### Conclusions

Understanding the potential for social media to improve health and well-being in Australian Aboriginal communities is important for researchers, public health professionals, and policy makers. Our scoping review found that there is potential for social media to provide a space for sharing health-promoting messages and increase awareness and self-efficacy of Aboriginal people in governing their own health and for social support. The cocreation of social media content involving Aboriginal people with the aim to improve health appears to influence participation when framed in a positive health context or form of self-empowerment. However, not all social media approaches are positively associated with Aboriginal people, and some negative health relationships still exist and require further exploration. There is a need for the development and implementation of cocreated messages with the Australian Aboriginal community delivered over social media and the subsequent measurement of its impact on health outcomes.

## Appendix

Multimedia Appendix 1 A summary of included studies on the impact of social media on Aboriginal health outcomes.
