# Supplementary material for: Considering the Impact of Social Media on Contemporary Improvement of Australian Aboriginal Health: Scoping Review
Source: JMIR Public Health Surveill. 2019 Feb 5;5(1):e11573. doi: 10.2196/11573 (PMC6379811; doi:10.2196/11573)
Supplement: Multimedia Appendix 1 [file publichealth_v5i1e11573_app1.docx]

**Multimedia Appendix 1.** A summary of included studies on the impact of social media on Aboriginal health outcomes.

| Author/Year | Study Aims | Population | Social Media | Method | Findings | Q |
| --- | --- | --- | --- | --- | --- | --- |
| Carlson et al (2015)  [21] | To conceptualise ideas surrounding Aboriginal deaths (Sorry Business) and funeral ceremonies alongside the use of social media in notifying families, offering support, grieving and healing. | Ages not specified | Facebook | Qualitative: Online interviews and perspectives from Aboriginal people. | Facebook was used to:  1. Provide notifications of funerals.  2. Offer condolences and extend support.  3. Grieve and heal.  Some relatively older Aboriginal people comparatively saw Facebook as detrimental regarding Sorry Business compared with younger Aboriginal people (ages not specified). | N |
| Carlson et al (2015) [23] | To gain insights into how Aboriginal people utilise and interact on social media, and how these technologies can assist with suicide prevention strategies. | Aged 18-60 years  Eight communities in NSW, SA, QLD and WA.  n=not stated | Facebook | Multiple Methods-  Qualitative: interviews (n=50)  Quantitative: survey (n=not stated)  Outlining emphasis on Aboriginal suicide rates and methods to indirectly or directly minimise progression/constancy relative to non-Aborignial people. Considers the ‘cultural context’ of Aboriginal suicide compared with non-Aboriginal suicide relative to health and well-being. | Facebook was used:  1. As an online support network - It was a positive, less invasive and daunting way to seek help and engage in two-way discussions about suicide prevention.  2. To reach a broader audience - Facebook provided opportunities to offer help with the possibility of reaching a greater/broader audience to provide those at risk with more help/support.  3. Suicide prevention strategies- Facebook offers a digital method via private messaging and checking in on individuals at potential risk of harm.  4. For rapid information dissemination- Facebook provided a quick and easy way to distribute information to a wide audience.  5. Mediating tragedy – Using Facebook may be detrimental if individuals feel they have a lack of knowledge in giving support and ‘advertising’ issues in a public space; This negative may be outweighed by greater monitoring and care towards those considering suicide or the use of functions like private messages to inhibit public advertisement concern. | P |
| Sweet et al (2013) [20] | To examine the themes of a day-long Twitter discussion about Aboriginal health and well being. | n=346 individuals  n=108 organisations | Twitter | Qualitative analysis of 423 active tweets recorded. | Twitter was used to communicate about health and wellbeing.  The most common tweets were about:  Social and emotional well-being (n=84 tweets)  Empowering (n=49 tweets)  Wider determinants of health and health issues (n=32 tweets)  Racism (n=28 tweets)  Advocacy and political engagement (n=25 tweets)  Cultural connections and health and identity and safety (n=36 tweets) | N |
| McPhail-Bell et al (2017) [11] | To understand collaborative ways social networking sites can work for health promotion agendas of social determinants and empowerment in an Aboriginal Australian context. | Ages not specified  South East Queensland | Facebook, Instagram, Twitter and YouTube -  A *Deadly Choices* health campaign | Qualitative: Ethnographic fieldwork observations | Identified five key principles underpinning the Deadly Choices campaign and betterment of Aboriginal health and wellbeing;  1. Create a dialogue  2. Build community online and offline. Combines offline positive activities/events and promotes them online for increased recognition.  3. Incentivise healthy online engagement  4. Celebrate Aboriginal identity and culture  5. Prioritise partnerships big or small.  Social networking sites can be used as a two-way communication to inspire, encourage and reinforce healthy (‘deadly’) behaviour changes within and between Aboriginal people. | G |
| Berends et al (2016) [22] | To encourage and support positive lifestyle change among the Gippsland Aboriginal community by promoting sporting role models, activities and events using social media. | Aged 16+ years  n=not stated  Gippsland, Victoria | Facebook, videos on Facebook and own interactive ‘Deadly Sport’ website | Multiple Methods-  Quantitative:  Web analytics from Facebook Insights  A community survey (n=47) about the #ChooseHealthy social marketing campaign run by Deadly Sport Gippsland  Qualitative:  interviews (n=3) with stakeholders from partner organisations in  Gippsland, who were also interviewed early in the evaluation | The Deadly Sport campaign used social media to host competitions, link to events and provide health information.  An overall increased number of followers and reach has been attained since inception of the program. Reported number of ‘likes’ on Facebook was 1,242 at October 28, 2015 and increased to 1,738 at May 2, 2016. 67% of respondents were women.  61% (n=20) of survey respondents had thought about changing their behaviour.  39% (n=13) of survey respondents reported actually changing their behaviour as a result of the campaign.  Combined messages and campaigns within the evaluation included topics like quitting smoking, decreasing consumption of added sugar and sugar sweetened beverages and encouraging physical activity. | P |

^a^*N*, Neutral.

^b^P, Poor.
